# Supplementary material for: Redox-dependent substrate-cofactor interactions in the Michaelis-complex of a flavin-dependent oxidoreductase
Source: Nat Commun. 2017 Jul 14;8:16084. doi: 10.1038/ncomms16084 (PMC5519977; doi:10.1038/ncomms16084)
Supplement: Supplementary Information [file ncomms16084-s1.pdf]

SI GUIDE

Type of file: pdf
Size of file: 7,869 KB
Title of file for HTML: Supplementary Information
Description: Supplementary Figures, Supplementary Tables and Supplementary References.

Type of file: pdf
Size of file: 293 KB
Title of file for HTML: Peer Review File
Description:

SUPPLEMENTARY FIGURES

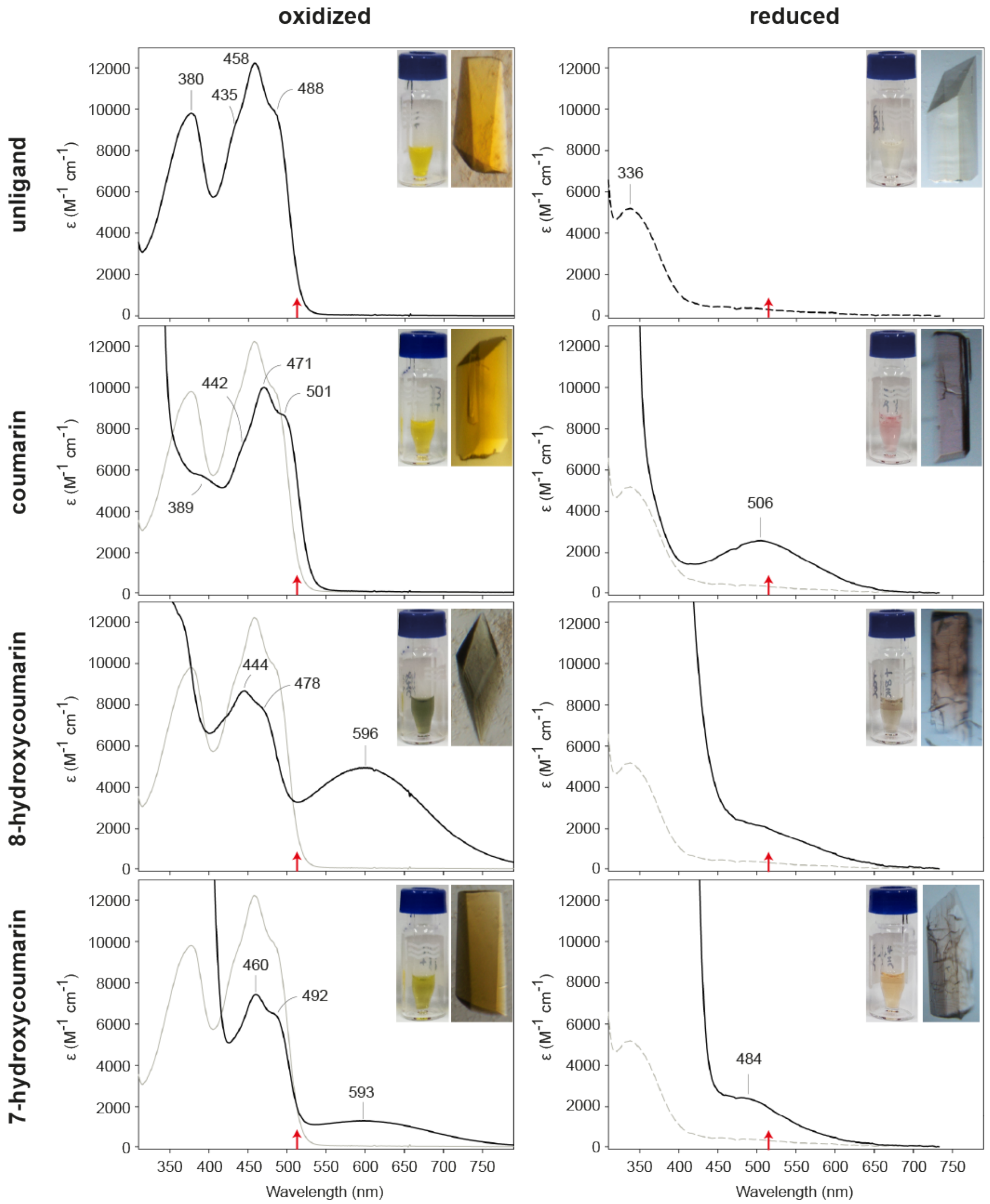

Supplementary Figure 1: UV/Vis spectra of different intermediates observed for the reactions catalyzed by XenA. The visible region of the spectra of the Y183F-XenA variant is shown. The spectra were recorded with an Agilent UV 8453 spectrophotometer (oxidized species) or a TIDAS photodiode array spectrophotometer coupled to a rapid mixing device (reduced species). Spectra of the reduced species were measured 3 ms (dead time of the experimental setup) after mixing the reduced enzyme with an excess of substrate. For a better comparison, the spectra of the unligated oxidized (solid gray line) or reduced (solid dashed line) XenA are overlaid with the spectra of the respective substrate complexes. Red arrows indicate the excitation wavelength using in resonance Raman experiments. The inserts show crystals and solutions of Y183F-XenA in complex with the corresponding oxidative substrates. The color change of the crystals upon substrate binding reveals formation of the non-productive Michaelis-complex mimic and authentic Michaelis-complex *in crystallo*.

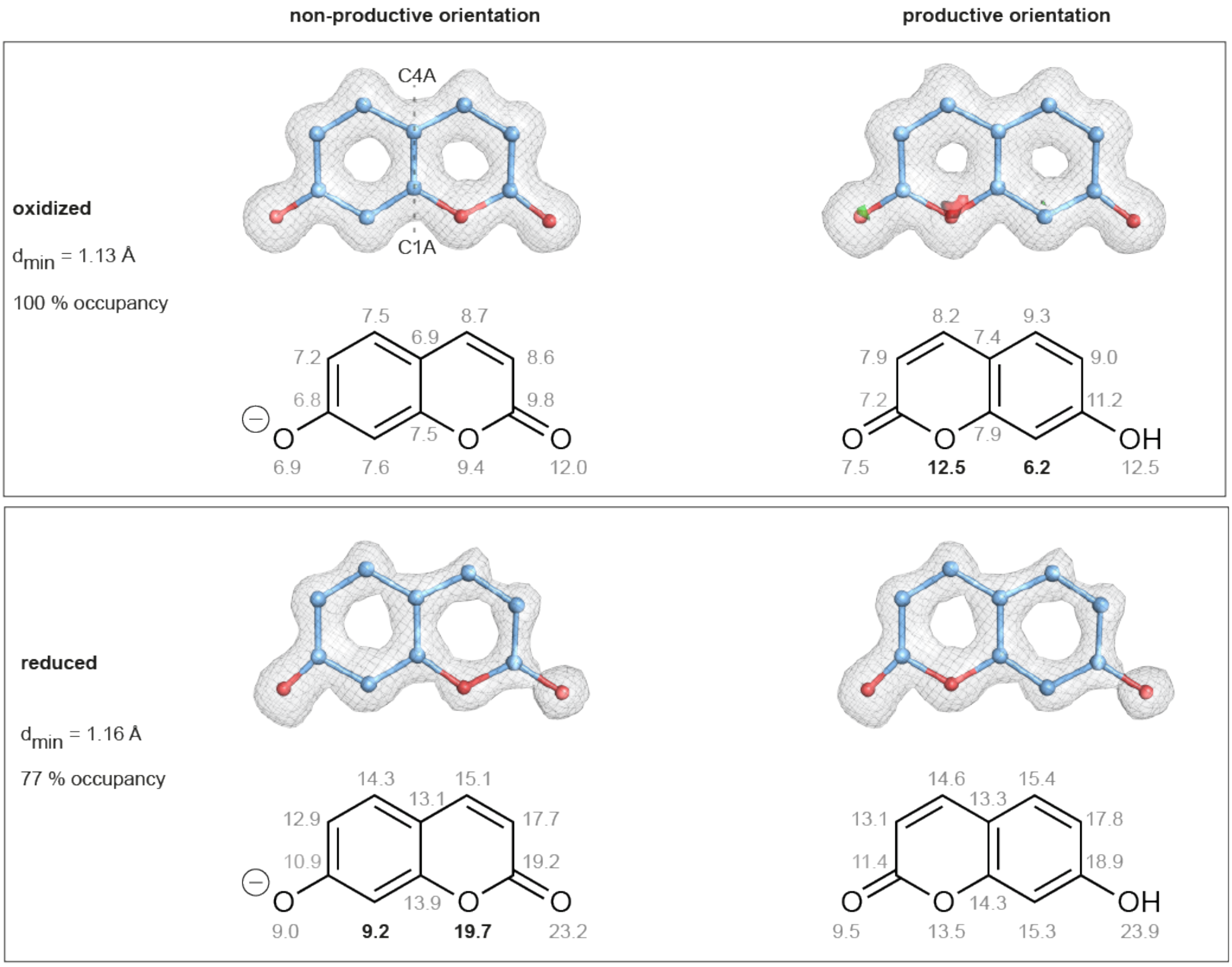

Supplementary Figure 2: Comparison of different binding modes of 7-hydroxycoumarin to the active site of Y183F-XenA in oxidized and reduced redox state. For both oxidation states the 2*F*o – *F*c and *F*o – *F*c maps for both orientations are contoured at 1.0 σ and 3.0 σ, respectively. The B-factor values (Å^2) of the individual atoms are shown in the chemical structure. As C and O differ only by two electrons, difference density was ambiguous for choosing the correct orientation. We therefore assume that the correct orientation should have a lower variation in B values of bonded atoms, especially at places where O and C may be swapped in the wrong orientation.

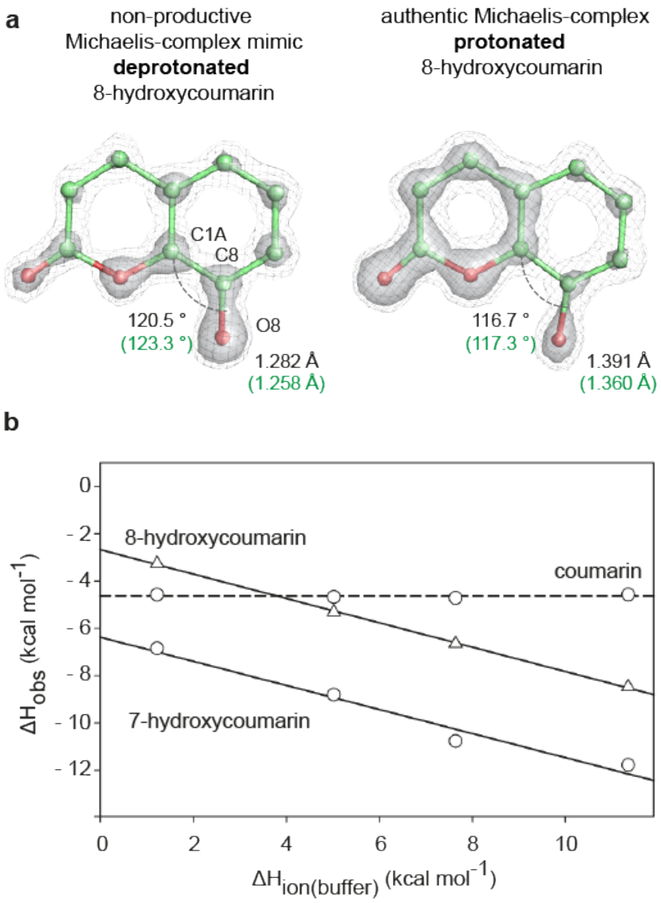

Supplementary Figure 3: Coupling between substrate binding and protonation. (**a**) Different protonation states of 8-hydroxycoumarin bound to the active site of oxidized and reduced Y183F-XenA variant. The 2*F*o – *F*c maps for the deprotonated and protonated state of 8-hydroxycoumarin are contoured at 2.5 σ and 1.5 σ, respectively. The surface presentation shows the 2*F*o – *F*c maps at 4.0 σ or 2.7 σ, respectively. The experimentally observed C8-O8 bond length and the C1A-C8-O8 angle agree well with the theoretical ones derived from DFT calculations (green numbers in parenthesis). Based on the DPI error estimator, the coordinate error for atoms with average B-factors is 0.03 Å [1]. (**b**) Proton linkage experiments for the binding of 8-hydroxycoumarin, 7-hydroxycoumarin and coumarin to Y183F-XenA. The observed enthalpy ΔH_{obs} for ligand binding is shown as a function of the deprotonation enthalpy ΔH_{ion} of the applied buffer. The slope of the linear regression yields the number of protons captured or released by the complex during the binding reaction. Experiments were performed as described in Material and Methods section with c-values ranging from 2 to 90, yielding slopes of -0.43 (8-hydroxycoumarin), -0.42 (7-hydroxcomarin) and close to zero (coumarin).

The negative slopes indicate that protons are released to the buffer upon binding of 7- or 8-hydroxycoumarin to Y183F-XenA, respectively. The release of “0.43” protons is in agreement with the Henderson-Hasselbalch-equation if the corresponding pKa-values for 7-hydroxycoumarin (7.8) and 8-hydroxycoumarin (8.0), and the chosen buffer pH (7.5) is used for calculation [2].

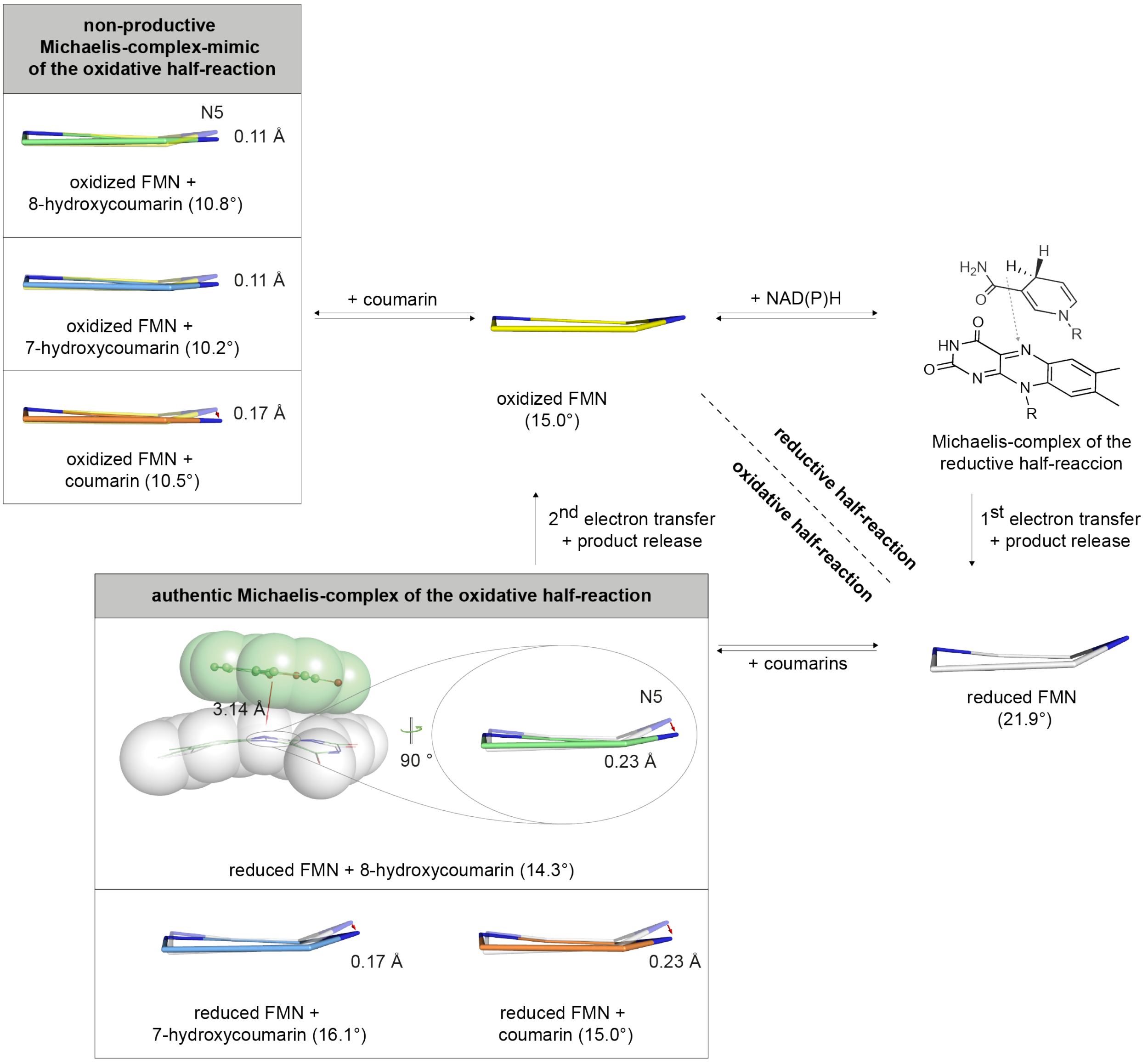

Supplementary Figure 4: Changes of the flavin geometry during the catalytic cycle of Y183F-XenA exemplified for the substrate 8-hydroxycoumarin. For each reaction intermediate, the pyrazine subnucleus of the isoalloxazine ring, after aligning the pyrazine and pyrimidine rings (excluding N5, N10, C2, and N3 atoms), is shown. Substrate binding sterically compresses the isoalloxazine ring, resulting in a decrease of the flavin butterfly-angle and in a displacement of the N5 atom. The observed structural changes are similar for all investigated substrates (8-hydroxycoumarin, 7-hydroxycoumarin and coumarin).

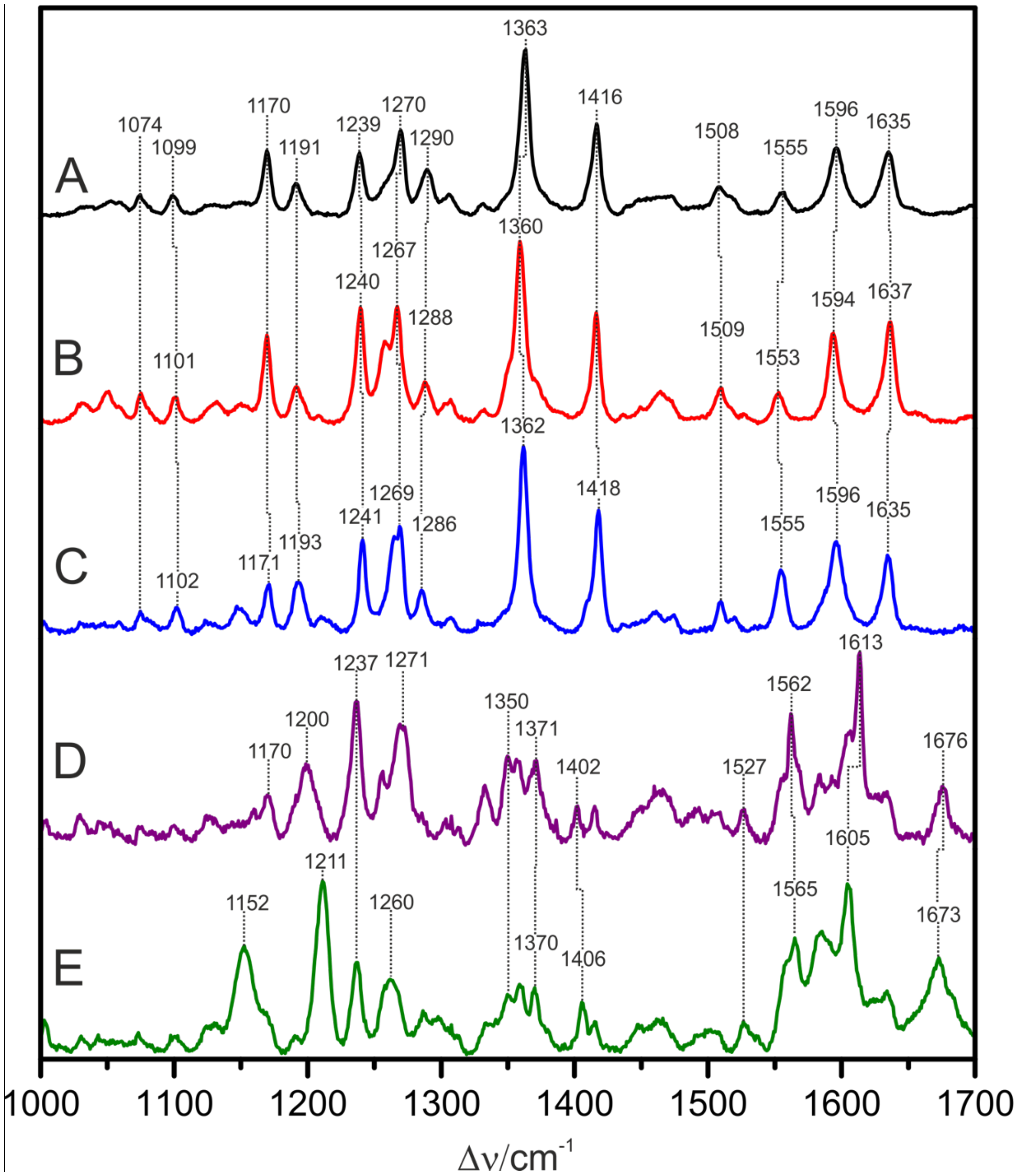

Supplementary Figure 5: RR spectra of oxidized and reduced Y183F-XenA in H_2O, measured with 514 nm excitation. A, XenA, oxidized; B, oxidized XenA-coumarin complex; C, oxidized XenA-7-hydroxycoumarin complex; D, reduced XenA-coumarin complex; E, reduced XenA-7-hydroxycoumarin complex.

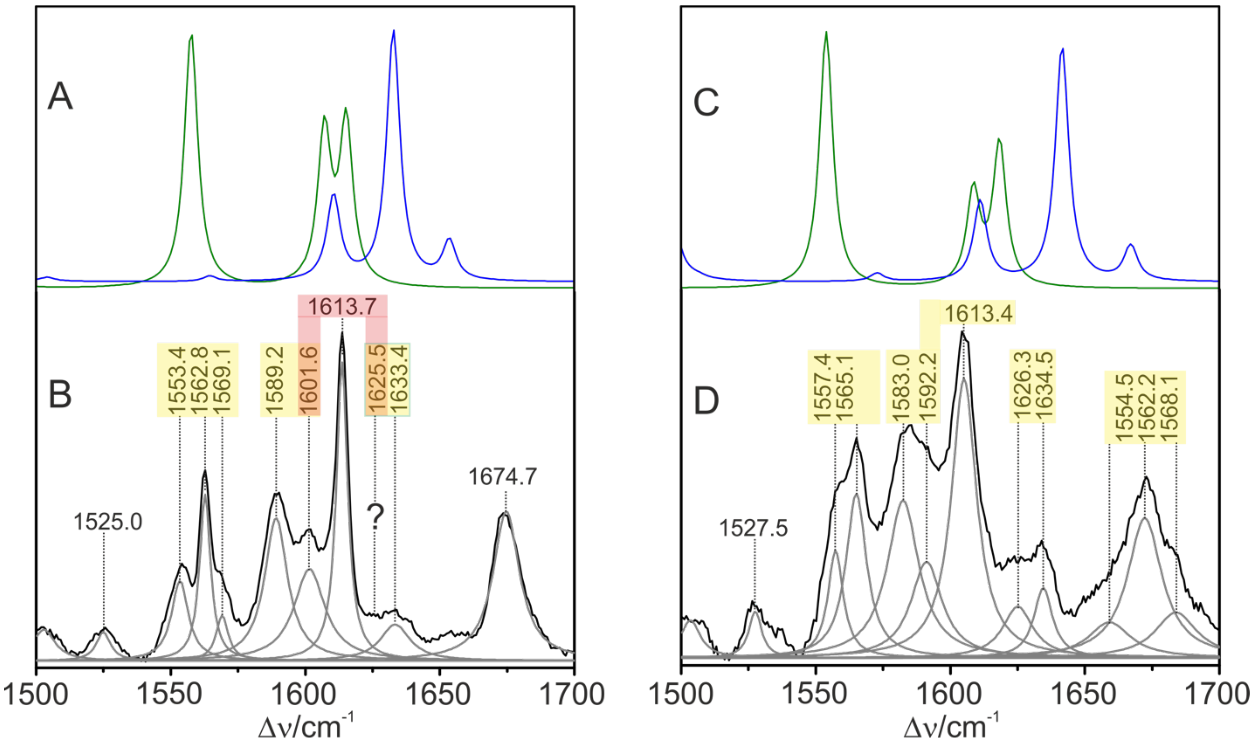

Supplementary Figure 6: RR spectra of reduced XenA substrate complexes measured with 514 nm excitation, compared with calculated spectra of cofactor and substrate radicals. Left: A, Calculated spectra of the reduced flavin (N-deuterated; blue trace) and coumarin (green trace); B, experimental RR spectrum of the reduced complex of Y183F-XenA and coumarin measured in D_2O. Right: C, Calculated spectra of the reduced flavin (blue trace) and 7-OH coumarin (green trace); D, experimental RR spectrum of the reduced complex of Y183F-XenA and coumarin measured with 514-nm excitation in H_2O. Yellow- and red-shaded peak labels indicate conjugate bands (triplets, doublets).

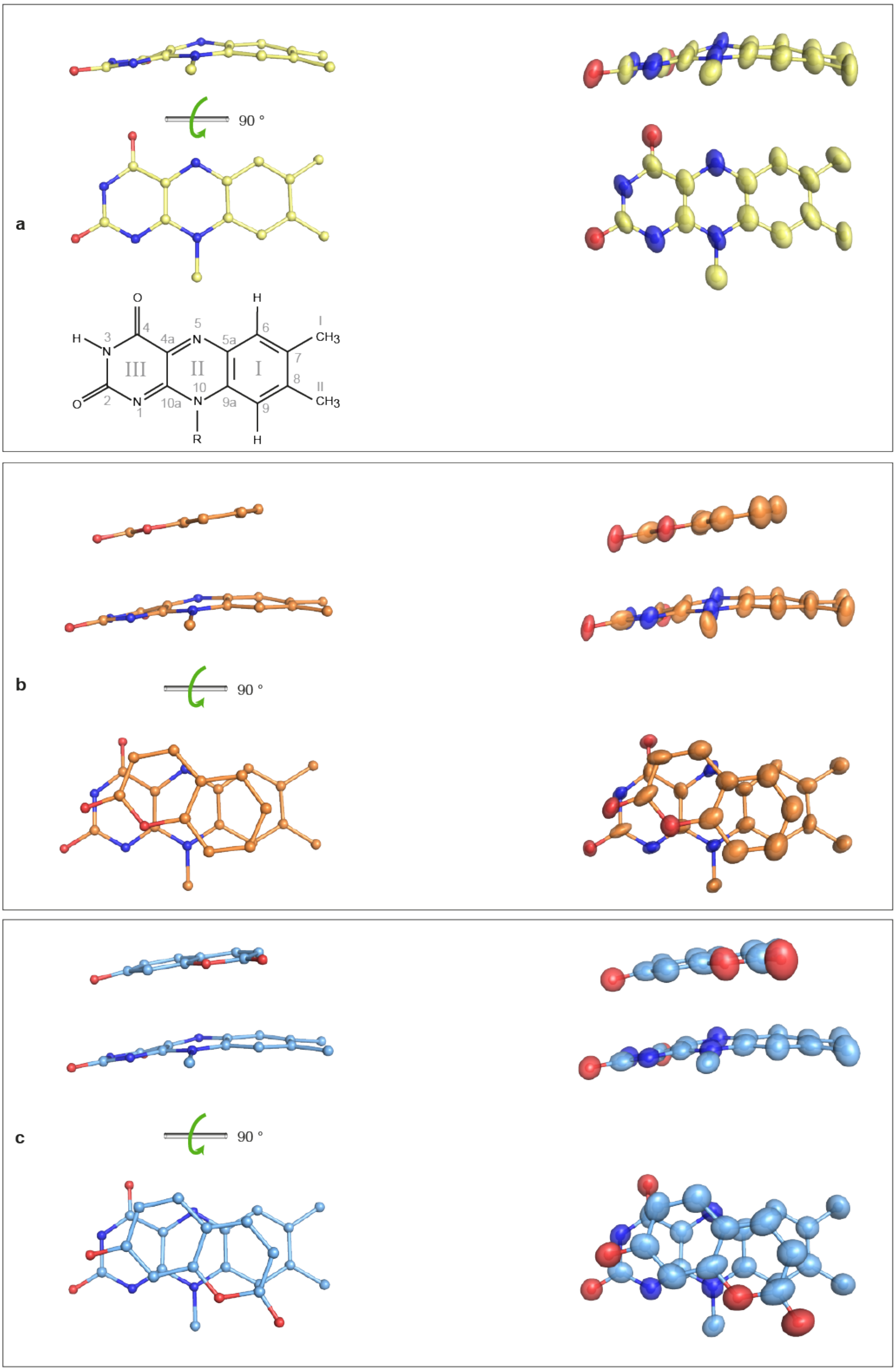

Supplementary Figure 7: Changes of the atomic displacement parameters upon substrate binding for oxidized Y183F-XenA in the absence of substrate (**a**) or in the presence of coumarin (**b**) and 7-hydroxycoumarin (**c**). The thermal ellipsoids are shown at the 50 % probability level.

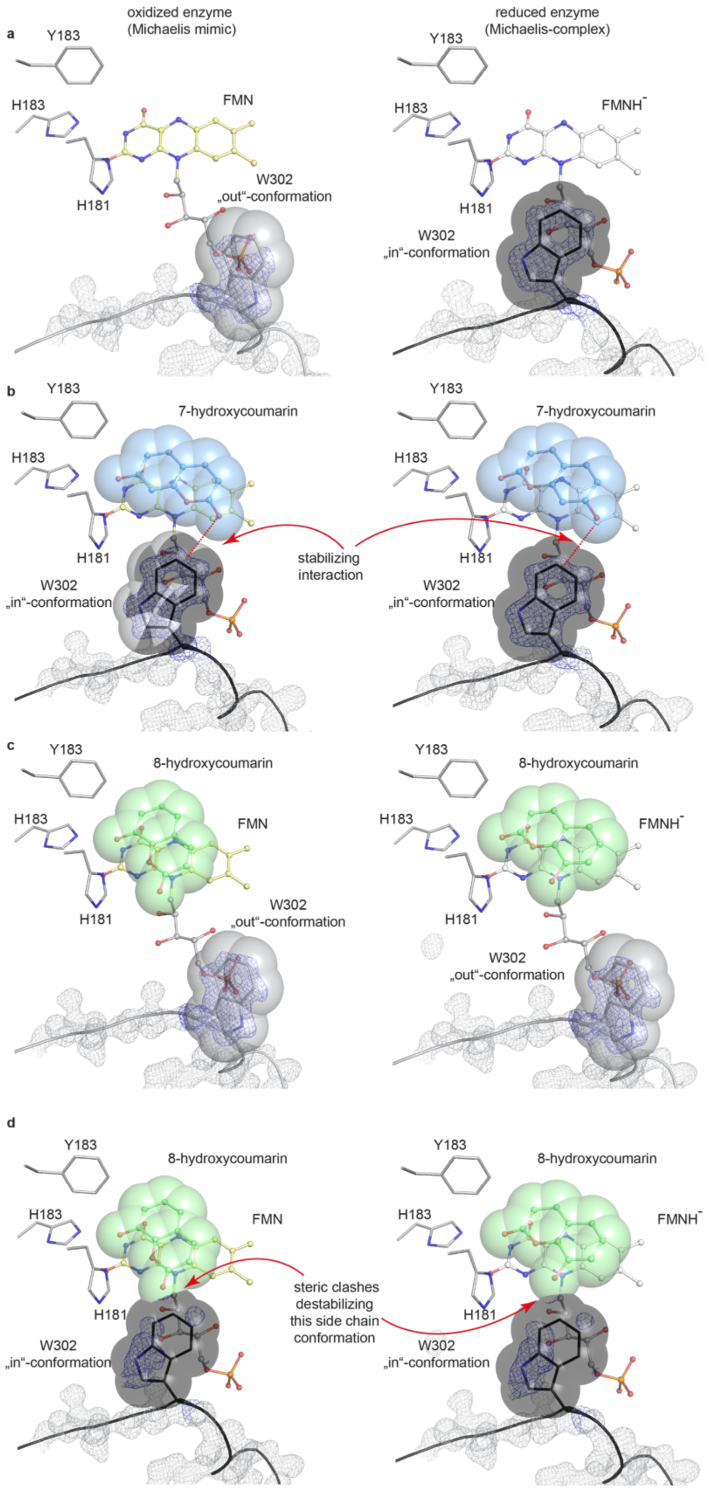

Supplementary Figure 8: Conformational changes of W302-XenA in response to the flavin oxidation state and substrate binding. (**a**) In the ligand-free enzyme, the W302 residue can adopt two conformations. The so-called "out-conformation" is highly populated in the oxidized enzyme, whereas the "in-conformation" is observed in the reduced enzyme. (**b**) W302 exhibits the "in-conformation" in the complex-structures with 7-hydroxycoumarin, regardless the redox-state. The conformation is stabilized by a C-H hydrogen bond interaction between the side chain and the substrate. (**c**) Contrary, the oxidized and reduced complexes with 8-hydroxycoumarin display the "out-conformation" of W302. (**d**) The "in-conformation" would result in steric clashes between the substrate and the W302 side chain, destabilizing the "in-" and stabilizing the "out-conformation". In all panels, spheres represent the van-der-Waals radii of the indole ring of W302 or the bound substrate, respectively. The Fo-Fc-omit maps of the loop region bearing W302 are contoured at 1 σ.

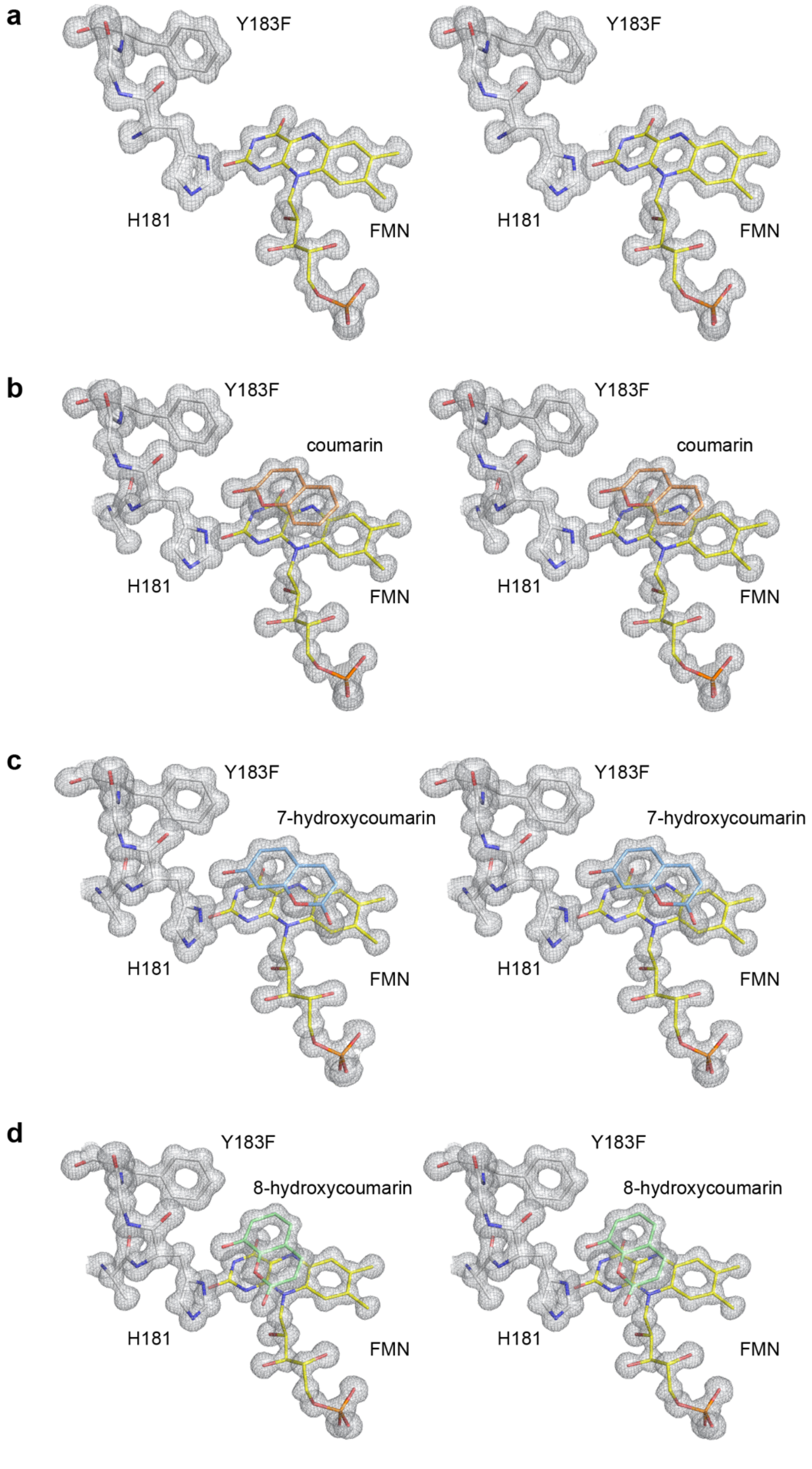
a
Y183F
H181
FMN
Y183F
H181
FMN
b
Y183F
coumarin
H181
FMN
Y183F
coumarin
H181
FMN
c
Y183F
7-hydroxycoumarin
H181
FMN
Y183F
7-hydroxycoumarin
H181
FMN
d
Y183F
8-hydroxycoumarin
H181
FMN
Y183F
8-hydroxycoumarin
H181
FMN

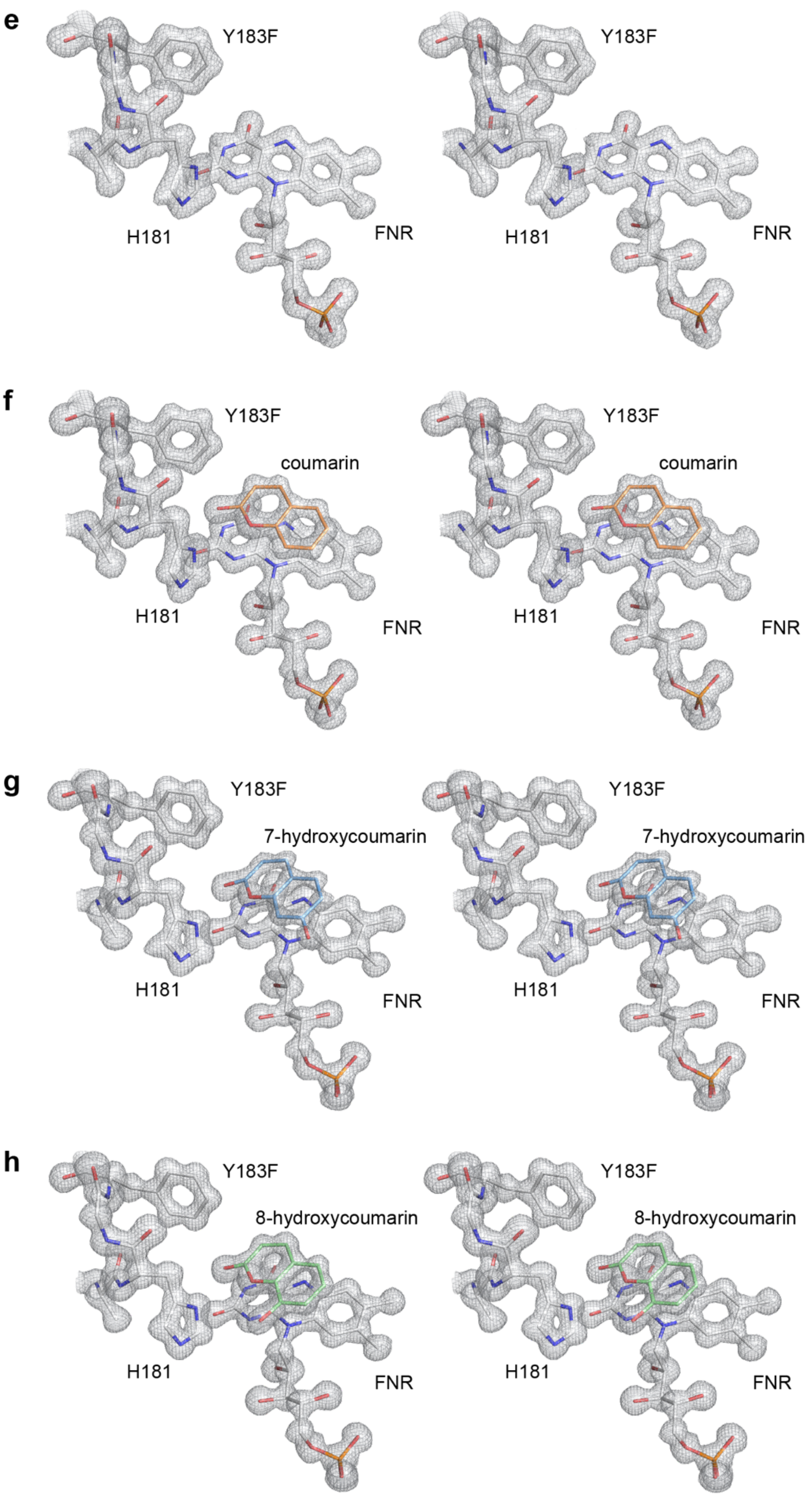

Supplementary Figure 9: Stereo images of the active site of Y183F-XenA with electron density. Oxidation states are in (**a-d**) oxidized and (**e-h**) reduced, either without ligand (**a, e**) or in complex with coumarin (**b, f**), 7-hydroxycoumarin (**c, g**) and 8-hydroxycoumarin (**d, h**). A representative portion of the $2F_o$-F_c electron density map for the active site is contoured at 1.5 σ and displayed in wall-eyed stereo.

SUPPLEMENATRY TABLES

Supplementary Table 1: Thermodynamic and kinetic constants for the reoxidation of wt-XenA and Y183F-XenA by coumarin, 7-hydroxycoumarin and 8-hydroxycoumarin. The oxidative half-reaction was measured in 50 mM Tris/HCl at pH 8.0 under anaerobic conditions at 25 °C. Dissociation constants (K_D) and limiting rate constants (k_{ox}) were derived by fitting the equation $k_{obs} = (k_{ox} [S])/(K_D + [S])$ to the data (± values are the fitting errors). Because the reoxidation of the Y183F variant by coumarin, 7-hydroxycoumarin and 8-hydroxycoumarin are very slow compared to the time-scale of a typical ITC experiment, the dissociation constants of the Michaelis-complexes were determined calorimetrically. The corresponding reoxidation rate constants for this variant are apparent rate constants in presence of 1.25 mM substrate.

	wt-XenA		Y183F-XenA	
substrate	K_D (μM) (SF)	k_{ox} (s^{-1})	K_D (μM) (ITC)	k_{ox} (s^{-1})
coumarin	23.3 ± 1.1	0.369 ± 0.004	37.2 ± 0.6	ND[a]
7-hydroxycoumarin	88.5 ± 4.1	0.083 ± 0.001	35.7 ± 1.7	$(980.1 \pm 25.2)\cdot 10^{-7}$
8-hydroxycoumarin	234 ± 15	0.681 ± 0.010	212 ± 16	$(897.2 \pm 9.6)\cdot 10^{-7}$

[a] ND – not detected under the applied conditions.

Supplementary Table 2: Data collection and refinement statistics for the uncomplexed as well as for the coumarin-, 7-hydroxycoumarin- and 8-hydroxycoumarin-bound structures of oxidized and reduced Y183F-XenA variant.

	**oxidized enzyme Michaelis-complex mimics of the oxidative half-reaction**				**reduced enzyme Michaelis-complexes of the oxidative half-reaction**			
**ligand**	**No**	**Cou**	**7-HC**	**8-HC**	**no**	**Cou**	**7-HC**	**8-HC**
**Data collection**								
Space group	*I* 2 2 2	*I* 2 2 2	*I* 2 2 2	*I* 2 2 2	*I* 2 2 2	*I* 2 2 2	*I* 2 2 2	*I* 2 2 2
Cell dimensions								
a, *b*, *c* (Å)	57.5, 83.2, 156.2	57.8, 83.4, 156.4	57.8, 83.4, 156.9	57.9, 83.4, 156.4	57.7, 83.5, 157.1	57.8, 83.5, 157.3	57.6, 83.5, 156.9	57.5, 83.4, 156.5
α, β, γ (°)	90, 90, 90	90, 90, 90	90, 90, 90	90, 90, 90	90, 90, 90	90, 90, 90	90, 90, 90	90, 90, 90
Resolution (Å)	29.24 – 1.25 (1.30 – 1.25)	38.73 - 1.10 (1.14 - 1.10)	35 - 1.13 (1.17 - 1.13)	29.28 - 1.07 (1.11 - 1.07)	28.84 - 1.44 (1.49 - 1.44)	36.89 - 1.23 (1.27 - 1.23)	27.04 - 1.16 (1.20 - 1.16)	20.15 - 1.09 (1.13 - 1.09)
R_{merge}	0.055 (0.62)	0.057 (0.53)	0.058 (0.41)	0.052 (0.54)	0.058 (0.58)	0.048 (0.61)	0.051 (0.57)	0.041 (0.35)
$I / \sigma I$	14.01 (1.96)	13.87 (2.56)	13.64 (3.22)	14.56 (2.54)	13.63 (2.25)	14.94 (2.60)	12.83 (2.39)	15.40 (2.74)
Completeness (%)	99.59 (98.13)	99.01 (96.74)	96.87 (91.60)	99.51 (99.59)	99.05 (99.54)	99.38 (98.19)	99.55 (99.65)	99.37 (96.86)
Redundancy	3.6 (3.5)	3.6 (3.5)	4.2 (3.6)	4.0 (3.4)	3.6 (3.5)	3.6 (3.6)	3.6 (3.5)	3.5 (2.6)
**Refinement**								
Resolution (Å)	29.24 – 1.25 (1.30 – 1.25)	38.73 - 1.10 (1.14 - 1.10)	35 - 1.13 (1.174 - 1.133)	35 - 1.07 (1.11 - 1.07)	28.84 - 1.44 (1.49 - 1.44)	36.89 - 1.23 (1.27 - 1.23)	27.04- 1.159 (1.200 - 1.159)	35 - 1.09 (1.13 - 1.09)
No. reflections	374906 (35574)	553670 (51381)	572508 (46114)	664946 (55916)	245543 (23550)	399414 (38273)	467856 (45181)	545123 (39287)
R_{work} / R_{free}	0.1358 (0.1642)	0.1249 / 0.1410	0.1182 / 0.1405	0.1226 / 0.1377	0.1464 / 0.1777	0.1206 / 0.1417	0.1263 / 0.1466	0.1197 / 0.1362
No. atoms								
Protein	2921	3005	3051	3055	2904	2954	3103	3027
Ligand/ion	41	57	81	58	46	90	63	53
Water	395	435	471	465	406	323	384	393
B-factors								
Protein	12.50	10.20	11.40	11.20	15.50	14.60	12.40	12.00
Ligand/ion	13.60	11.10	17.90	11.90	18.30	21.40	16.90	12.20
Water	28.30	26.30	25.60	25.10	30.80	25.30	28.00	27.20
R.m.s. deviations								
Bond lengths (Å)	0.009	0.010	0.010	0.010	0.011	0.009	0.010	0.010
Bond angles (°)	1.27	1.38	1.39	1.40	1.22	1.32	1.40	1.43

*Values in parentheses are for highest-resolution shell

Supplementary Table 3: Experimental frequencies of the oxidized flavin cofactor of Y183F-XenA and calculated frequencies of isolated riboflavin *in vacuo*.[a]

experimental		calculated						
H_2O	D_2O	no.	H_2O	PED	no.	D_2O	PED	ring
1635	1636	24	1623	ν C(9)-C(8) 12%<br>ν C(6)-C(7) 28%<br>ν C(5a)-C(6) 11%	24	1623	ν C(9a)-C(9) 10%<br>ν C(9)-C(8) 12%<br>ν C(6)-C(7) 28%<br>ν C(5a)-C(6) 11%	I
1596	1597	25	1555	ν C(4a)-N(5) 23%<br>ν C(5a)-C(9) 12%<br>ν C(9)-C(8) 10%	25	1555	ν C(4a)-N(5) 23%<br>ν C(5a)-C(9) 12%<br>ν C(9)-C(8) 10%	II
1556	1556	26	1523	ν C(7)-C(8) 10%<br>ν N(1)-C(10a) 10%	26	1524	ν C(7)-C(8) 10%	I, II
1508	1508	27	1506	ν N(1)-C(10a) 34%<br>ν C(4a)-N(5) 16%	27	1507	ν N(1)-C(10a) 35%<br>ν C(4a)-N(5) 16%	II
1416	1416	36	1421	ν C(4a)-N(5) 11%	36	1421	ν C(4a)-N(5) 11%	II
1363	1365	43	1358	δ N(3)-H 12%<br>ν C(5a)-C(9a) 11%	43	1357	ν C(5a)-C(9a) 12%	I, II, III
1305	1307	48	1319	ν N(10)-C(10a) 14%<br>ν C(9a)-C(9) 12%<br>ν N(3)-C(4) 11%	47	1320	ν N(10)-C(10a) 15%<br>ν C(9a)-C(9) 12%	I, II
1289	–	51	1279	δ ring I 10%<br>δ ring II 17%<br>ν N(5)-C(5a) 15%	50	1280	δ ring I 10%<br>δ ring II 17%<br>ν N(5)-C(5a) 15%	I, II
1269	1272	52	1257	δ C(9)-H 29%<br>δ C(6)-H 13%	51	1257	δ C(9)-H 28%<br>δ C(6)-H 12%	I
1238	1239	53	1250	ribitol > 44%	52	1250	ribitol > 43%	
–	1216	–	–	–	54	1239	ν N(3)-C(4) 30%<br>ν C(2)-N(3) 23%<br>δ N(3)-H 12%	III
1191	1191	60	1169	δ C(6)-H 15%<br>ν N(3)-C(4) 19%	–	–	–	I, III
1169	1171	61	1137	δ ring I 10%	60	1138	ν N(3)-H 11%<br>δ ring I 10%	I, III
–	1150	–	–	–	62	1117	ν N(1)-C(2) 15%<br>δ N(3)-H 15%<br>δ C(4)=O 10%	III
1098	–	62	1129	ν C(8)-methyl 15%<br>ν C(7)-methyl 15%<br>δ ring I 10%	–	–	–	I
–	1082				65	1071	ribitol > 39%	
1074	–	70	1023	ν N(10)-ribitol 15%	–	–	–	II

[a] Frequencies are given in cm^{-1}; notations: ν, stretching coordinate; δ, bending coordinate; “ribitol” refers to the coordinates of the ribitol residue; PED, potential energy distribution; for atom and ring numbering see Supplementary Fig. 7a.

Supplementary Table 4: Frequencies and full-widths at half height of the band components in the RR spectra of reduced XenA complexes as derived a band fitting analysis.

XenA-coumarin, H_2O		XenA-coumarin, D_2O		XenA-7-hydroxycoumarin, H_2O		XenA-7-hydroxycoumarin, D_2O	
ν/cm^{-1}	$\Delta\nu/cm^{-1}$	ν/cm^{-1}	$\Delta\nu/cm^{-1}$	ν/cm^{-1}	$\Delta\nu/cm^{-1}$	ν/cm^{-1}	$\Delta\nu/cm^{-1}$
1505.2	12.6	1502.9	9.9	1503.4	8.9	1505.1	13.0
1526.4	4.8	1525.0	6.4	1527.5	5.8	1529.9	4.8
1554.5	5.9	1553.4	7.6	1557.4	6.9		
1562.2	5.5	1562.8	4.9	1565.1	9.3	1558.8	7.5
1568.1	6.7	1569.1	5.5				
1583.0	8.1	1589.2	10.3	1582.5	14.3	1585.6	17.8
1592.2	8.8			1591.2	14.0	1593.9	8.1
1604.5	12.0	1601.6	13.9	1605.1	11.3	1605.0	13.7
1613.4	5.3	1613.7	5.1				
1626.3	8.3			1625.1	13.7	1623.1	20.5
1634.0	6.5	1633.4.	13.4	1634.5		1635.1	8.0
				1659.2	19.0		
1675.7	9.2	1674.7	12.0	1672.3	15.5	1673.1	13.3
				1683.9	18.9	1683.2	3.1

Supplementary references

1. Blow, D.M. Rearrangement of Cruickshank's formulae for the diffraction-component precision index. *Acta Crystallogr D Biol Crystallogr* **58**, 792-7 (2002).
2. Baker, B.M. & Murphy, K.P. Evaluation of linked protonation effects in protein binding reactions using isothermal titration calorimetry. *Biophysical Journal* **71**, 2049-2055 (1996).
